# Printed Thermoresistive Carbon and PEDOT:PSS Temperature Sensors on Different Substrates

**DOI:** 10.3390/s26216903

**Published:** 2026-10-31

**Authors:** Cláudia S. Buga, Júlio C. Viana

**Affiliations:** 1 IPC/LASI—Institute for Polymers and Composites, Associated Laboratory of Intelligence Systems, University of Minho, Campus de Azurém, 4800-058 Guimarães, Portugal; claudia.buga@dtx-colab.pt; 2 DTx Colab—Digital Transformation Collaborative Laboratory, University of Minho, Campus de Azurém, 4800-058 Guimarães, Portugal

**Keywords:** PEDOT:PSS, temperature sensors, positive temperature coefficient, negative temperature coefficient, substrates

## Abstract

Printed thermoresistive sensors are strongly influenced by the sensing material, substrate, printing process and encapsulation. In this work, PEDOT:PSS temperature sensors were fabricated by inkjet printing on PET and glossy paper, while carbon-based sensors were produced by screen printing on the same substrates. Their morphology, chemical and thermal characteristics, electrical properties, impedance response and thermoresistive performance were systematically compared. PEDOT:PSS exhibited a pronounced substrate dependence: sheet resistance increased from 57.93 ± 13.88 Ω/sq on PET to 5804.75 ± 1732.37 Ω/sq on paper, accompanied by a change from PTC behaviour on PET to NTC behaviour on paper. TCR values changed from +0.176 ± 0.012% °C^−1^ on PET to −0.594 ± 0.049% °C^−1^ on paper. In contrast, carbon sensors retained PTC behaviour on both substrates, with TCR values of +1.168 ± 0.093% °C^−1^ on PET and +1.002 ± 0.097% °C^−1^ on glossy paper in the unencapsulated condition. Encapsulation reduced the magnitude of the PEDOT:PSS response on both substrates, while for carbon, this effect was directly assessed only on glossy paper, where TCR decreased from +1.002 ± 0.097 to +0.510 ± 0.014% °C^−1^. The results demonstrate that the combined ink–substrate–printing-process system is decisive in determining the temperature response of printed sensors.

## 1. Introduction

Printed temperature sensors are attracting increasing interest for flexible, lightweight, and low-cost monitoring systems in areas such as wearable electronics, robotics, biomedical devices, smart packaging and environmental sensing [1]. Recent work has even highlighted substantial advances in sensitivity, flexibility, response time, stability, stretchability, and integration into wearable and implantable platforms [2,3,4]. Many of these applications involve moderate temperature ranges, and comparable operating windows have been widely investigated for printed thermoresistive sensors intended for wearable and smart-packaging applications. Despite this progress, the response of printed thermoresistive sensors is still strongly dependent on the complete material–substrate–process system. In particular, comparatively less attention has been given to cases in which the same conductive material exhibits markedly different electrical characteristics, or even a change in the sign of the temperature coefficient, as a consequence of substrate and processing conditions.

Thermoresistive sensors usually comprise a temperature-sensitive electrically conductive material, connected between two electrodes, which is deposited over a supporting substrate [5]. Variations in temperature alter the electrical resistance of the active material, allowing temperature to be estimated from the corresponding resistance–temperature response. The sensitivity of thermoresistive sensors is commonly expressed through the temperature coefficient of resistance (TCR), which can be obtained from the slope of the normalised resistance variation with temperature:

(1) $$\mathrm{TCR}\left(\%{}^{\circ}C^{-1}\right)=100\times \frac{d(∆R/R_{0})}{\mathrm{dT}},$$

where ∆*R* = *R*(*T*) − *R*_0_, *R*_0_ is the initial resistance at temperature *T*_0_, and *R*(*T*) is the electrical resistance of the sensor at temperature *T*. A positive TCR corresponds to a positive temperature coefficient (PTC) response, in which resistance increases with temperature, whereas a negative TCR corresponds to a negative temperature coefficient response (NTC), where electrical resistance decreases as temperature increases. The sign and magnitude of the TCR depend on the dominant electrical-transport mechanism [6]. Other sensor design parameters of interest include linearity, repeatability, hysteresis, response stability and compatibility with the intended operating range [7].

In metallic conductors, resistance typically increases with temperature because enhanced lattice vibrations increase electron–phonon scattering and reduce the mean free path of charge carriers, resulting in a PTC response [8]. However, in semiconducting and disordered conductive materials, increasing temperature can instead facilitate carrier activation, hopping, or tunnelling, frequently leading to an NTC response [9]. In conductive polymer composites, thermomechanical effects may also contribute: differences in the coefficients of thermal expansion (CTE) of the polymeric matrix, conductive phase, and supporting substrate can alter the separation between conductive domains or particles, disrupt conductive pathways and consequently, modify the temperature-dependent resistance [10,11].

The thermoresistive behaviour of printed sensors cannot, therefore, be determined solely by the intrinsic properties of the sensing material. It is also strongly influenced by the substrate, electrode configuration, printing technology and resulting layer morphology [12]. Flexible polymeric substrates such as polyethylene terephthalate (PET), polyethylene naphthalate (PEN), polyimide (PI), thermoplastic polyurethane (TPU), polydimethylsiloxane (PDMS), paper, textiles, and glass have been thoroughly investigated as platforms for temperature-sensor development [13]. These substrates differ substantially in surface roughness, porosity, surface energy, moisture affinity and CTE. As a result, the same conductive ink may exhibit different spreading, penetration, drying, film continuity, electrical conductivity and thermoresistive behaviour, depending on the substrate on which it is deposited.

The printing technology further influences the structure and properties of the sensing layer. Inkjet printing enables digital, maskless and material-efficient deposition of low-viscosity inks and is particularly suited to the fabrication of thin patterned films, whereas screen printing is compatible with more viscous pastes and generally produces thicker conductive layers with higher material loading [13]. The combined selection of ink, printing technology and substrate is therefore critical for determining the morphology, the electrical properties and the temperature-sensing performance of printed thermoresistive devices.

Among the conductive materials used for printed temperature sensing, poly(3,4-ethylenedioxythiophene):polystyrene sulfonate (PEDOT:PSS) has received considerable attention because of its electrical conductivity, mechanical flexibility, biocompatibility, water-solution processability and compatibility with several printing and coating methods [14]. It is a conducting polymer complex composed of positively charged PEDOT chains and negatively charged, electrically insulating PSS-rich regions surrounding or separating a PEDOT-rich conductive domain [15,16]. In practice, PSS balances the positive charges on PEDOT, enabling its processing as a stable aqueous dispersion. The abovementioned advantages make PEDOT:PSS particularly attractive for inkjet-printed flexible electronics and have motivated its extensive investigation as a temperature-sensitive material, both in its pristine form and in composites containing conductive fillers such as carbon nanotubes, graphene, graphene oxide and metallic particles [17,18,19]. Different sensor geometries have also been reported, including straight conductive strips, meander patterns and films deposited over interdigitated electrodes; these are further detailed in Table 1 [5,16,18,19,20,21,22,23,24,25].

As presented in Table 1, most PEDOT:PSS-based temperature sensors exhibit an NTC response, generally attributed to thermally assisted charge transport between localized PEDOT-rich domains. In disordered films, this behaviour is commonly described using variable-range hopping, although tunnelling, weak localization and electron–electron interactions may also contribute [9]. The hygroscopic PSS-rich phase can further influence the response through moisture uptake, swelling, hydrogen bonding and changes in interdomain spacing [17]. Bali et al. [19] showed that DMSO addition progressively reduced the magnitude of the negative TCR of inkjet-printed PEDOT:PSS sensors, with the response approaching approximately −0.25% °C^−1^ at higher DMSO concentrations. The same study also demonstrated that encapsulation markedly improved sensor stability under damp-heat conditions, highlighting the strong influence of environmental exposure on PEDOT:PSS-based sensors. In another study, Barmpakos et al. reported an inkjet-printed meander-shaped PEDOT:PSS temperature sensor on glossy paper exhibiting an NTC response with a TCR of approximately −1.39% °C^−1^ over 25–45 °C, illustrating the influence of paper-based substrates on PEDOT:PSS thermoresistive behaviour [20].

PTC responses have also been reported, with the temperature dependence of PEDOT:PSS being substantially modified through processing and secondary doping. Choi et al. [26] demonstrated that DMSO doping can induce a transition from hopping-dominated to more band-like charge transport, accompanied by substantial suppression and eventual change in the temperature dependence of electrical resistance. Hinckley et al. [21] similarly associated metallic-like transport and positive TCR with improved PEDOT-chain alignment after solution shearing. Vuorinen et al. [18] observed a change from NTC under argon to PTC under ambient conditions, highlighting the influence of humidity, while Yu et al. [27] achieved a TCR of up to +4.2% °C^−1^ in PEDOT:PSS/PDMS sensors by introducing pre-strain-induced microcrack openings during substrate expansion. In our previous work, inkjet-printed PEDOT:PSS sensors on PET also exhibited a PTC response, with TCR values of approximately +0.18% °C^−1^ for non-encapsulated sensors and +0.075% °C^−1^ after encapsulation [16]. Overall, these studies and the ones compiled in Table 1 show that the thermoresistive response of PEDOT:PSS is strongly influenced by formulation, morphology, environmental conditions, processing and thermomechanical interactions with the substrate or conductive fillers.

Carbon-based materials constitute another important class of printed thermoresistive sensors [28]. Their electrical transport generally depends on direct contacts and tunnelling between neighbouring conductive particles or agglomerates, while thermal expansion of the polymeric binder or substrate can alter particle separation and junction resistance [29]. Consequently, carbon-based sensors may exhibit either NTC or PTC behaviour, depending on the carbon allotrope, filler concentration, binder composition, substrate, and device architecture. Literature studies illustrate this variability. For instance, Lee et al. [30] reported an NTC response for CNT/epoxy temperature-sensitive coatings over −20 to 60 °C, and Huber et al. [31] demonstrated a screen-printed carbon-black-based temperature-sensor array, with TCR values ranging from −0.50 to −3.60% °C^−1^. In contrast, Ali et al. [32] reported a positive TCR of approximately +0.375% °C^−1^ for a carbon-black sensing layer deposited over printed silver electrodes on a flexible polyimide substrate. In another previous work, screen-printed commercial carbon inks on PET also exhibited PTC behaviour, with TCR values reaching approximately +1.55% °C^−1^ [5]. Together, these studies also highlight the strong influence of the conductive carbon network, polymeric matrix, substrate and fabrication method on thermoresistive behaviour.

**Table 1:** Comparison of PEDOT:PSS and carbon-based temperature sensors from the literature.

Active Material	Substrate	Method	Design	TCR (% °C^−1^)	Range (°C)	Ref.
PEDOT:PSS	Glossy paper	Inkjet printing	Meander	−1.39	25–45	[20]
PEDOT:PSS	Glossy paper	Inkjet printing	Strip	−3.00	−10–22	[33]
PEDOT:PSS	PET	Inkjet printing	IDE	−0.80	20–70	[24]
PEDOT:PSS	PET	Spin coating	Meander	−0.72	27–100	[26]
PEDOT:PSS	PET	Spin coating	Meander	+0.09	27–100	[26]
PEDOT:PSS	PI	Inkjet printing	Strip	+0.113	28–90	[23]
PEDOT:PSS	PDMS	Drop casting	IDE	+4.2	30–55	[27]
PEDOT:PSS	Polyamide textile	Inkjet printing	Meander	−0.47	30–50	[34]
PEDOT:PSS	PET	Inkjet printing	Meander	+0.075 encapsulated +0.18 non-encapsulated	25–60	[16]
PEDOT:PSS (w/DMSO) 2 Carbon inks	PEN	Shadow mask	Rectangle (Wheatstone bridge)	−0.25 (PEDOT:PSS) +0.22, +0.10 (carbon)	20–70	[19]
Graphene/PEDOT:PSS	Polyurethane	Inkjet printing	Meander	−0.06 (argon) +0.07 (ambient)	34–44	[18]
CNT/Epoxy	Epoxy composite/coating	Mold/Solution casting	Square (array)	−0.07	−20–60	[30]
CB composite	PEN	Screen printing/Aerosol jet	Square (array)	−0.50 to −3.60	5–90	[31]
Graphene/PDMS	PDMS	3D printing	Cellular structure	+0.80	25–75	[35]
Commercial carbon inks	PET	Screen printing	Meander	+1.12 (CNP ink)/+1.46 (CNT-type ink)	25–75	[5]

In summary, both PEDOT:PSS-based and carbon-based temperature sensors can display either NTC or PTC responses. The literature therefore suggests that temperature sensitivity cannot be attributed to the sensing material alone, but rather to the combined effects of material formulation, substrate, morphology, sensor design, environmental exposure and processing conditions. Building on these observations, the present study investigates how substrate, conductive ink, printing technology and encapsulation affect the performance of printed thermoresistive temperature sensors. PEDOT:PSS sensors were fabricated by inkjet printing on PET and glossy paper, whereas carbon-based sensors were produced by screen printing on the same substrates. Their structural, electrical and thermoresistive properties were compared to clarify how the combined ink–substrate–printing-process system influences temperature sensitivity and sensor behaviour. Accordingly, the novelty of the present work lies not in proposing a new class of flexible temperature sensor, but in systematically comparing how conductive ink, substrate, printing process, and encapsulation jointly affect the electrical and thermoresistive behaviour of printed sensors. Particular attention is given to the pronounced substrate-dependence of inkjet-printed PEDOT:PSS, including the transition from PTC behaviour on PET to NTC behaviour on glossy paper and to the contrasting substrate-independent PTC response of thicker screen-printed carbon layers.

## 2. Materials and Methods

### 2.1. Materials

Two printing inks were used in this work: (i) a water-based PEDOT:PSS ink (ORGACON^TM^ IJ-1005, from Agfa, Mortsel, Belgium) for inkjet printing and (ii) a carbon ink (Bectron GP 9553, Elantas Europe GmbH, Hamburg, Germany) for screen printing, with their main properties shown in Table 2.

**Figure 1:**
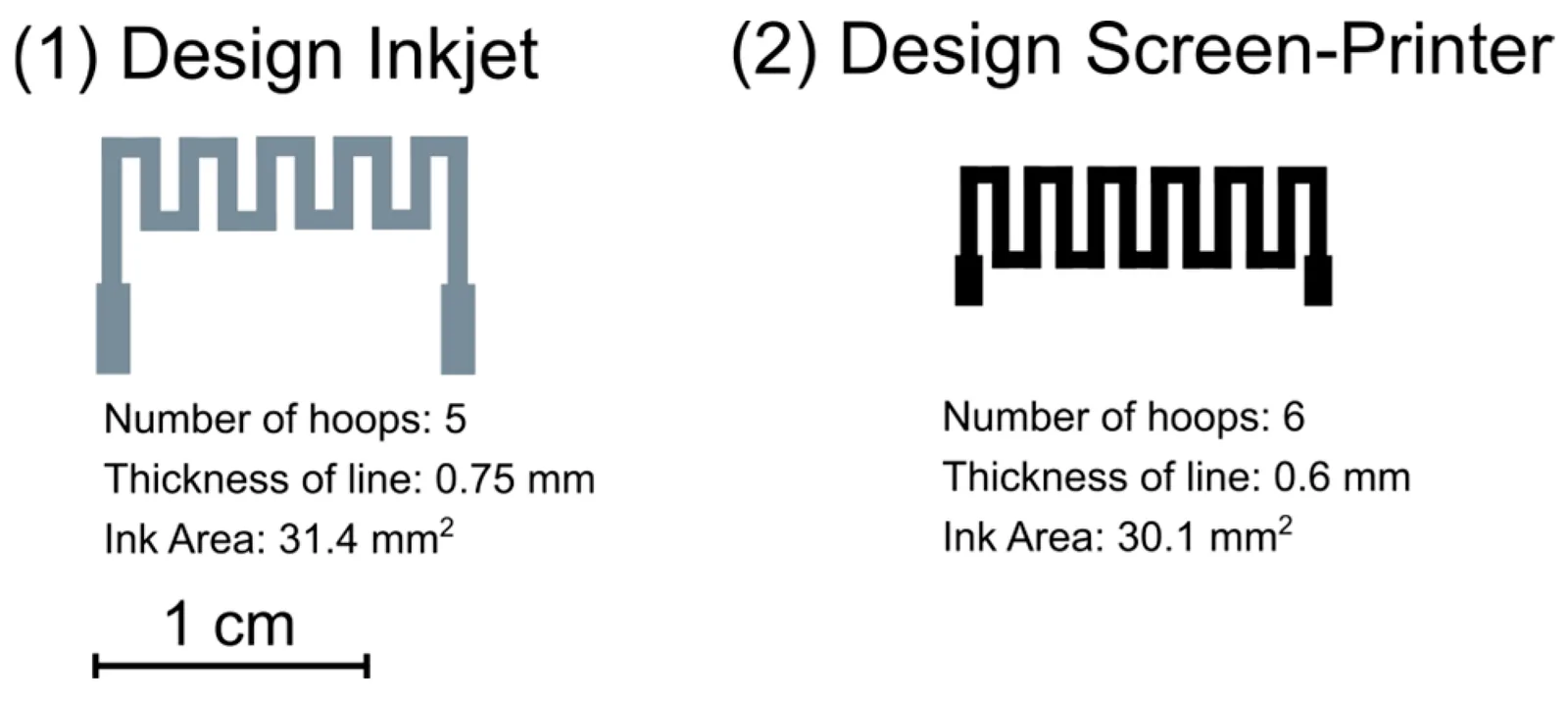
Meander-like sensor design (dimensions in mm).

Two substrates were selected: (i) a polyethylene terephthalate (PET) film with 150 µm thickness (from SK Chemicals Co., Ltd, Seongnam, Republic of Korea) and (ii) a glossy paper with 190 µm thickness (from Seiko Epson Corporation, Nagano, Japan; 216 g.m^−2^). For inkjet printing, the substrates were treated before printing using an atmospheric plasma equipment (Plasma APC 500, Diener Electronic GmbH & Co. KG, Ebhausen, Germany). A polyimide tape (150 µm) was used for sensor encapsulation. In all cases, a conductive silver ink pen (from Chemtronics, Kennesaw, GA, USA) was used to print the electrodes. The sensor designs were meander-like, as shown in Figure 1.

These designs were selected after previous optimisation work [5,16]. Table 3 describes the printed sensors produced and evaluated in this work.

### 2.2. Methods

#### 2.2.1. Printing Parameters

A Xennia Carnelian inkjet printer (Xennia Technology Ltd., Letchworth, UK), equipped with a QS Sapphire 256/AAA-10 printhead (Fujifilm Dimatix, Santa Clara, CA, USA), was used to fabricate the PEDOT:PSS temperature sensors (meander 1). Prior to printing, both PET and paper substrates were subjected to atmospheric plasma treatment using an APC 500 system. The plasma discharge was applied for 30 s at a distance of 7 cm from the substrate surface. Three successive printing passes were performed, with a 5 min interval between passes to allow for partial drying and stabilisation of the deposited ink. The printhead height and velocity were set to 1 mm and 60 mm s^−1^, respectively, while the maximum waveform voltage was 21 V. During printing, the substrate bed was maintained at 40 °C. After deposition, the printed patterns were cured at 90 °C for 20 min in a ventilated box oven.

The carbon-based temperature sensors (meander 2) were produced using a manual screen printer (Miniprint from Acosgraf, Vila Nova de Gaia, Portugal). In this case, no plasma pre-treatment was required. The mesh of the screen used to print the carbon sensors was made from SEFAR^®^ PME polyester mesh, type 71/48Y, where 71 refers to the mesh count (number of openings by inch square area of the screen), 48 μm is the diameter of the screen’s threads and Y relates to the yellow colour of the screen thread. A single printing pass was performed, followed by curing at 110 °C for 20 min in a ventilated box oven. Finally, silver electrodes were manually applied to the extremities of all the printed sensor patterns to enable electrical characterisation.

#### 2.2.2. Chemical and Morphological Characterisation

Representative photographs of the printed sensors were acquired to document the overall geometry, print definition, substrate appearance and encapsulation state. Surface topography, area roughness and printed-layer step height were evaluated using a Profilm3D optical profilometer (Filmetrics, San Diego, CA, USA) equipped with a 10× objective. Areal roughness was determined on the unprinted PET and glossy-paper substrates using the S_a_, S_q_ and S_z_ parameters, corresponding to the arithmetic mean height, root mean square height and maximum surface height, respectively. Printed-layer thickness was evaluated from step-height measurements across identifiable printed/unprinted boundaries. For the thin PEDOT:PSS films, these measurements were treated as apparent step-height estimates because optical transparency and the small film thickness limit the reliability of absolute thickness determination by optical profilometry.

The morphology and local elemental composition of the PEDOT:PSS- and carbon-printed patterns on the different substrates were further analysed by scanning electron microscopy (SEM) coupled with energy-dispersive X-ray spectroscopy (EDS) using a NanoSEM FEI Nova 200 system (FEI Company, Hillsboro, OR, USA).

The thermal stability and degradation behaviour of the PEDOT:PSS and carbon inks were evaluated by thermogravimetric analysis using a Q500 instrument (TA Instruments, New Castle, DE, USA), with heating from 100 °C to 600 °C under nitrogen atmosphere. Fourier-transform infrared (FTIR) spectroscopy was subsequently performed using a Spectrum 100 spectrometer (PerkinElmer, Waltham, MA, USA) allowing to identify the characteristic functional groups of the printed materials and to investigate possible chemical differences associated with the substrate and printing process.

#### 2.2.3. Electrical and Thermoresistive Characterisation

The sheet resistance of the printed conductive materials was preliminarily evaluated using a four-point probe system (Ossila Ltd., Sheffield, UK). For these measurements, approximately 10 × 10 mm square films of PEDOT:PSS and carbon ink were prepared on PET and glossy-paper substrates by drop/blade coating, respectively. Square specimens were used to provide a sufficiently large and geometrically well-defined area for positioning the four-point probe and entering the sample dimensions into the measurement software. The actual sensor geometries were not suitable for four-point probe characterisation because the conductive tracks were narrow and patterned, while the inkjet-printed PEDOT:PSS layers were extremely thin and difficult to contact reproducibly with the probe head. The measurements were therefore performed on dedicated unpatterned specimens prepared from the same inks and substrates. Sheet-resistance values were obtained directly from the four-point probe measurements, thereby minimizing the contribution of probe–sample contact resistance. Two independently prepared specimens were evaluated for each ink–substrate combination, and repeated readings were collected at different positions or during consecutive measurements on each specimen. The resulting sheet-resistance values were subsequently compared with the room-temperature resistance of the corresponding patterned sensors to assess whether the electrical trends observed in the unpatterned films were also reflected at the device level. Because the four-point-probe specimens were prepared separately, differences in film thickness and morphology may exist relative to the actual printed sensor tracks. Thus, these measurements were used primarily to compare relative ink–substrate trends rather than to quantitatively predict device resistance.

Electrical impedance spectroscopy (EIS) was performed using a PalmSens4 potentiostat (PalmSens BV, Houten, The Netherlands) to investigate whether the different ink–substrate combinations exhibited frequency-dependent electrical contributions beyond their DC resistance, particularly those potentially associated with substrate interaction, interfacial effects, moisture or encapsulation. Measurements were conducted in a two-electrode configuration using a sinusoidal perturbation of 10 mV at 0 V DC bias. Frequency sweeps were performed logarithmically, generally from 1 Hz to 100 kHz, with approximately 12 points per decade. For the lower-resistance carbon and PEDOT:PSS/PET devices, measurements could also be extended to higher frequencies in the initial characterisation, whereas the highly resistive PEDOT:PSS/paper devices showed increasing current-range underload and high-frequency dispersion, limiting reliable quantitative interpretation of the upper-frequency region. For these samples, repeated measurements were therefore performed over 1 Hz–100 kHz, while the predominantly resistive low- and intermediate-frequency region was considered for quantitative comparison. The current range was selected according to the sensor impedance; the repeated PEDOT:PSS/paper measurements were acquired using the 1 µA range, although the very low AC currents generated by these high-resistance devices resulted in an underload indication. To assess repeatability, multiple consecutive spectra were acquired without altering the electrical contacts. Impedance magnitude (∣Z∣), real impedance (Z′), imaginary impedance (−Z′′), and phase angle were evaluated and compared with the room-temperature DC resistance of the corresponding sensors.

**Figure 2:**
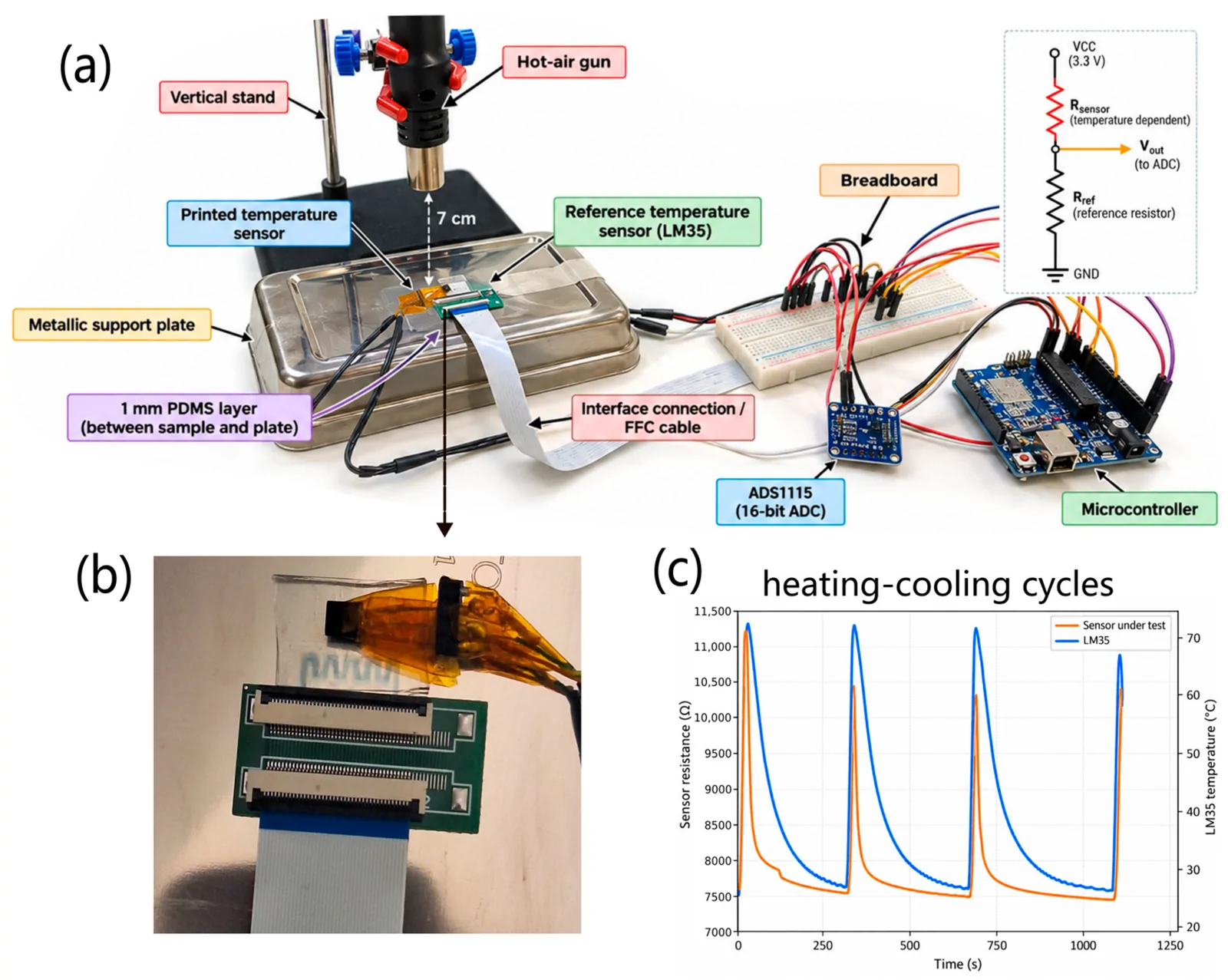
Experimental setup used for thermoresistive characterisation of the printed temperature sensors. (**a**) Overall measurement configuration and components; the inset shows the voltage-divider circuit. (**b**) Detail of the printed sensor and LM35 reference sensor positioned in close proximity during testing, i.e., on top of the PDMS interlayer. (**c**) Representative repeated heating–cooling cycles showing the sensor resistance and LM35 temperature response (extracted from an experiment conducted on a carbon/PET encapsulated sensor). Quantitative TCR analysis was performed using the heating branch only.

For continuous resistance acquisition during thermal testing, the sensor electrodes were connected to an Arduino-based acquisition system through a flexible printed-circuit extension cable. The acquisition circuit incorporated an ADS1115 16-bit analog-to-digital converter (Adafruit), as illustrated in Figure 2. A commercial LM35 temperature sensor was simultaneously connected to the system and positioned in close proximity to the printed sensor to provide the reference temperature. Each printed sensor was then incorporated into a voltage-divider circuit. The reference resistor was selected according to the expected resistance range of the sensor to improve measurement sensitivity, with lower-value resistors used for the lower-resistance devices and higher-value resistors used for the higher-resistance devices. Thus, a 10 kΩ reference resistor was used for the lower-resistance PEDOT:PSS/PET sensors and carbon-based sensors, whereas 100 kΩ or 330 kΩ reference resistors were used for the higher-resistance PEDOT:PSS/paper sensors.

The samples were heated using the hot-air source of a soldering station equipped with a circular nozzle approximately 2.1 cm in diameter, positioned at a fixed distance of 7 cm above the sensing region. The sensor was placed on an aluminium support plate, with a 1 mm PDMS interlayer positioned between the back surface of the sensor and the aluminium plate to provide a reproducible thermal-contact condition and promote more uniform heat distribution. The experimental geometry, illustrated in Figure 2, was maintained constant for all measurements. Since the setup relies on transient heating, the results should be interpreted primarily as standardised comparative measurements rather than as high-accuracy absolute temperature calibration.

For carbon/PET devices, thermoresistive characterisation was performed only after encapsulation, since preliminary heating cycles of unencapsulated devices resulted in progressive loss of adhesion and fracture of the printed carbon layer. Encapsulation was therefore required to ensure mechanical integrity during repeated thermal cycling (Table 3). Polyimide tape was selected as a practical flexible encapsulation layer to provide mechanical protection and environmental isolation; however, it was not intended as a thermally optimised encapsulation material.

Before each test, the initial resistance of the printed sensor was recorded at room temperature. Then, the heat source was switched on, and the temperature was progressively increased from approximately 25 °C to 70 °C. This temperature interval was selected as a moderate operating range suitable for comparative evaluation of the printed sensors on both PET and paper substrates. Comparable temperature ranges have also been reported for printed thermoresistive sensors intended for applications such as wearable electronics and smart packaging. The upper limit was restricted to approximately 70 °C to minimize thermomechanical changes of the PET support that could influence the printed conductive layer and its interface with the substrate.

After the target temperature was reached, the hot-air source was switched off, and the system was allowed to cool naturally toward its initial temperature. Four consecutive heating–cooling cycles were performed for each sensor, with printed-sensor resistance and LM35 temperature recorded simultaneously. Although complete heating–cooling cycles were acquired, quantitative thermoresistive analysis was restricted to the heating branch. The thin printed sensing layers exhibited a faster thermal response than that of the packaged LM35 reference sensor during cooling (Figure 2c), resulting in an apparent thermal lag. Consequently, the cooling branch was not used for TCR determination or quantitative hysteresis analysis. TCR, linearity, and cycle-to-cycle repeatability were therefore extracted from the heating response only. For each heating cycle, the resistance response was normalised to the resistance measured at the beginning of that cycle, *R*_0_, and expressed as ∆*R*/*R*_0_ (%). TCR was obtained from the slope of a linear regression fitted to the normalised resistance–temperature data over the heating branch, while the corresponding coefficient of determination (R^2^) was used to assess linearity. The values reported for each sensor type were derived from repeated heating cycles of three independently fabricated sensors, as detailed in the statistical analysis procedure. Cycle-to-cycle repeatability was assessed from the standard deviation of the TCR values obtained from the repeated heating cycles for each sensor.

For each sensor type, three independently fabricated sensors were evaluated, with four consecutive thermal cycles recorded per sensor. TCR and R^2^ were calculated from the heating branch of each cycle. In cases where the first cycle exhibited a clear stabilisation behaviour and increased the cycle-to-cycle variability above 10%, this initial cycle was treated as a conditioning cycle and excluded from the quantitative averaging. No subsequent cycles were excluded. The remaining cycle values were averaged for each sensor, and the final mean ± standard deviation was calculated across the three independent sensors. The resulting resistance–temperature curves were used to evaluate the temperature coefficient of resistance, calculated according to Equation (1), as well as linearity and cycle-to-cycle repeatability.

## 3. Results and Discussion

### 3.1. Morphological Characterisation of Printed Designs

**Figure 3:**
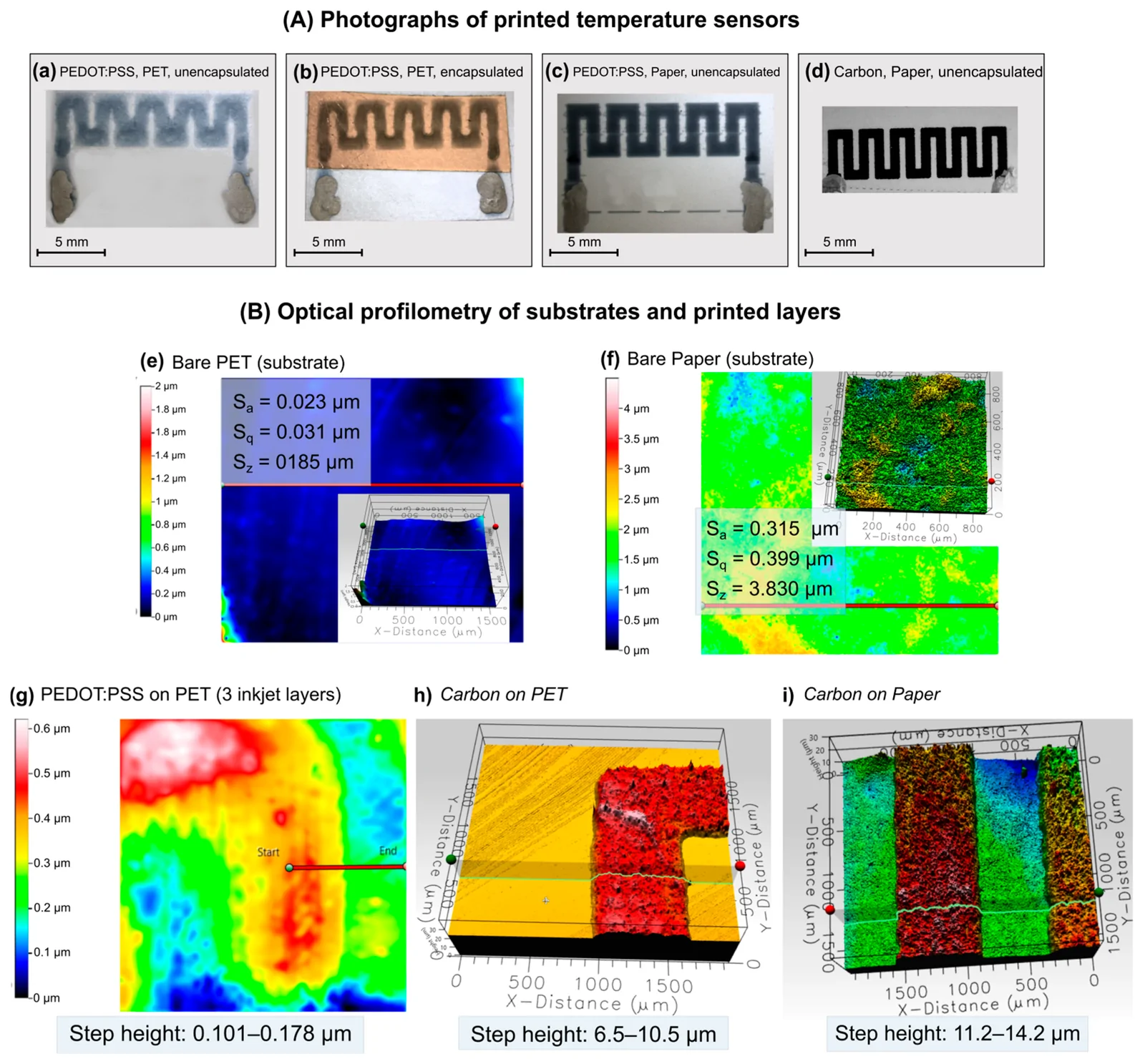
Morphological characterisation of the printed temperature sensors. (**A**) Photographs of: (**a**) PEDOT:PSS on PET, unencapsulated; (**b**) PEDOT:PSS on PET, encapsulated; (**c**) PEDOT:PSS on paper, unencapsulated; and (**d**) carbon on paper, unencapsulated. (**B**) Optical profilometry of: (**e**) bare PET substrate; (**f**) bare paper substrate; (**g**) PEDOT:PSS printed on PET after three inkjet printig passes; (**h**) screen-printed carbon layers on PET and (**i**) screen-printed carbon layers on paper. Roughness parameters: S_a_ (arithmetic mean height), S_q_ (root mean square height), and S_z_ (maximum surface height).

Representative photographs of the fabricated temperature sensors and optical profilometry measurements of the substrates and printed layers are presented in Figure 3A. The photographs confirm the successful fabrication of PEDOT:PSS sensors on PET and paper, as well as carbon-based sensors on paper, with clearly defined meander geometries. The encapsulated PEDOT:PSS/PET sample additionally illustrates the final configuration used to isolate the printed sensing layer from the surrounding environment.

Optical profilometry revealed substantial differences between the two substrates (Figure 3B). Bare PET exhibited a comparatively smooth surface, with S_a_ = 0.023 µm, S_q_ = 0.031 µm, and S_z_ = 0.185 µm, whereas glossy paper presented considerably higher roughness, with S_a_ = 0.315 µm, S_q_ = 0.399 µm, and S_z_ = 3.830 µm. This difference is particularly relevant for the measurement of the thin inkjet-printed PEDOT:PSS, since the surface-height variations of the paper are considerably larger than the expected thickness of the polymer layer. As a result, for PEDOT:PSS deposited on PET, the printed/unprinted boundary could be identified, allowing apparent step-height thickness values of approximately 0.10–0.18 µm to be estimated after three printing passes. However, these values should be interpreted cautiously as apparent optical step heights rather than as definitive absolute film thicknesses. On glossy paper, the substrate roughness and porosity exceeded the expected PEDOT:PSS layer thickness, preventing reliable thickness determination. In contrast, the screen-printed carbon layers were substantially thicker and could be clearly resolved on both substrates. Apparent step heights of approximately 6.5–10.5 µm were obtained on PET, increasing to approximately 11.2–14.2 µm on paper. The larger apparent thickness on paper may result from differences in ink deposition, spreading and interaction with the rougher and more porous substrate. Overall, the profilometry results confirm the markedly different layer dimensions produced by inkjet-printed PEDOT:PSS and screen-printed carbon, while also demonstrating the strong influence of substrate topography on the reliability of thin-film thickness measurements.

**Figure 4:**
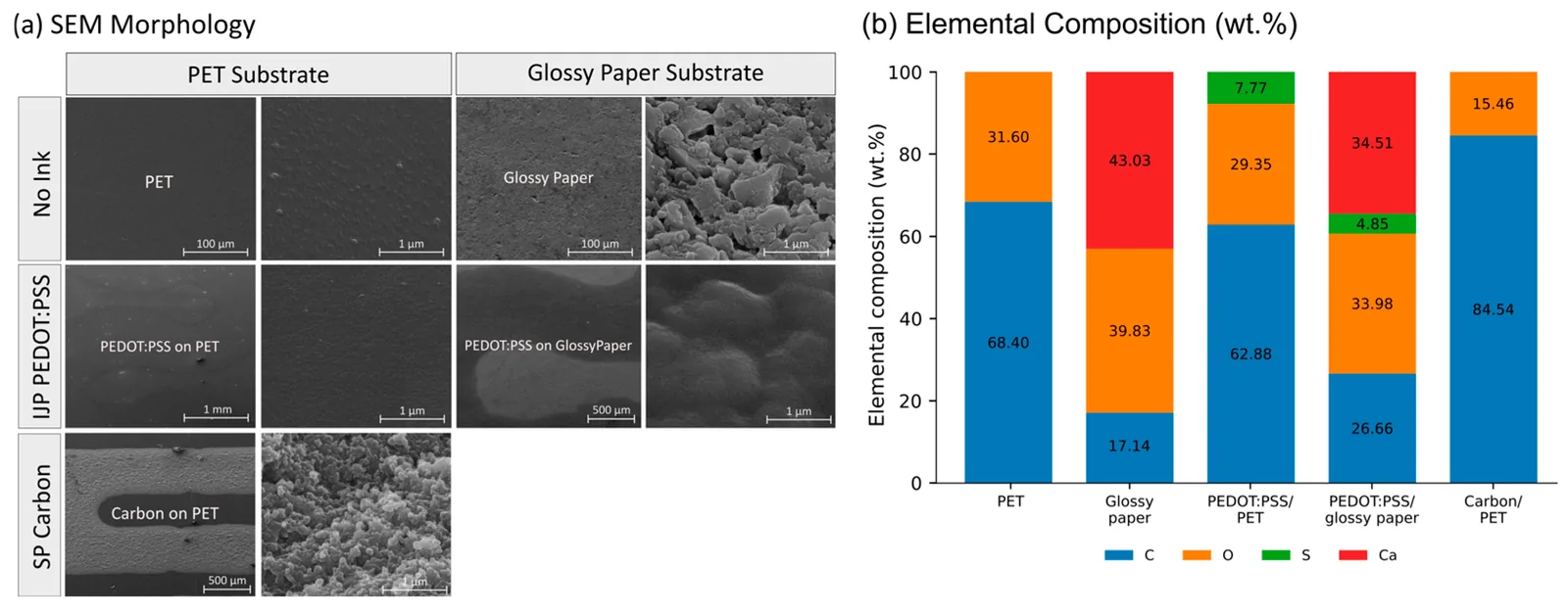
(**a**) Morphological and elemental characterisation of the substrates and printed conductive layers. (**a**) SEM images of bare PET and glossy paper substrates, inkjet-printed PEDOT:PSS on PET and glossy paper, and screen-printed carbon on PET, shown at different magnifications. (**b**) EDS elemental composition (wt.%) obtained from representative regions of bare PET, bare glossy paper, PEDOT:PSS/PET, PEDOT:PSS/paper, and carbon/PET.

SEM images comparing the surface morphology of the bare substrates and printed conductive layers are presented in (Figure 4a). In line with what was reported above, bare PET presents a relatively smooth and homogeneous surface, whereas glossy paper exhibits a considerably rougher and more heterogeneous morphology. These substrate differences remain evident after PEDOT:PSS deposition. The PEDOT:PSS layer printed on PET appears relatively smooth and continuous, while PEDOT:PSS printed on paper follows the underlying surface topography, resulting in a substantially rougher and less uniform morphology. This observation is consistent with the greater roughness and porosity of paper identified by optical profilometry. On the other hand, the screen-printed carbon layer on PET exhibits a markedly different morphology from that of the inkjet-printed PEDOT:PSS films, with a granular and particulate surface composed of submicrometric carbon-rich agglomerates. This morphology is consistent with the higher solids content and particulate nature of screen-printable carbon formulations and with the substantially thicker layer produced by screen printing compared with inkjet-printed PEDOT:PSS.

The EDS analysis further highlights differences between the substrates and printed layers (Figure 4b). Bare PET is predominantly composed of carbon and oxygen, whereas glossy paper additionally presents a strong calcium contribution, consistent with the presence of calcium-containing mineral fillers commonly used in white coated papers. Following PEDOT:PSS deposition on PET, sulphur is detected, along with carbon and oxygen, confirming the presence of the sulphur-containing PEDOT:PSS layer. In PEDOT:PSS printed on paper, sulphur is also detected, while a substantial calcium signal from the underlying substrate remains visible. The persistence of the substrate-related calcium signal is consistent with a thin and/or heterogeneous PEDOT:PSS layer and with the interaction of the ink with the paper or its partial penetration into the porous paper surface. Finally, the carbon layer printed on PET is dominated by carbon, with a smaller oxygen contribution, as expected for the carbon-based conductive formulation.

### 3.2. Thermal and Chemical Characteristics of the Inks

**Figure 5:**
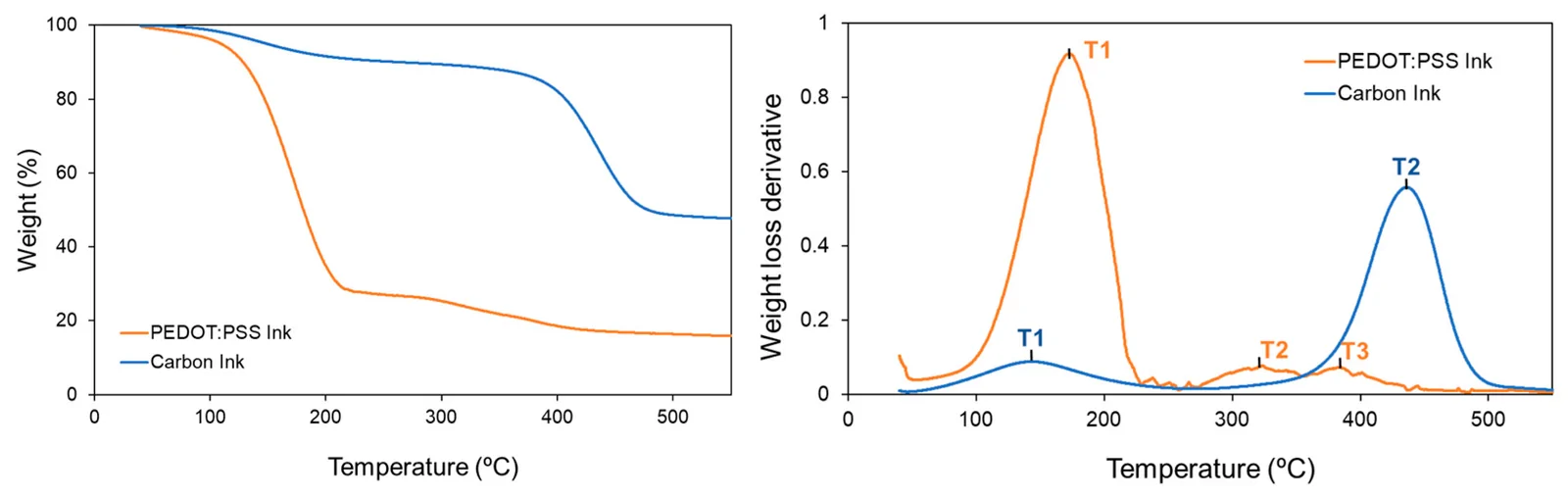
TGA plots of PEDOT:PSS and carbon inks.

Figure 5 compares the thermogravimetric behaviour of the PEDOT:PSS and carbon inks. The two formulations exhibit markedly different degradation profiles, reflecting their distinct compositions. The PEDOT:PSS ink shows a pronounced mass-loss event centred at approximately 174.8 °C, followed by two smaller degradation processes at approximately 320.3 and 382.4 °C. The first event is likely associated predominantly with the removal and decomposition of residual volatile components and formulation additives, whereas the higher-temperature processes can be associated with degradation of the PSS-rich phase and the subsequent decomposition of the PEDOT-containing polymeric structure. At 550 °C, approximately 15% of the initial mass remained.

The carbon ink exhibits substantially greater thermal stability. A relatively small degradation event occurs at approximately 141.8 °C, followed by the main mass-loss process centred at 436.2 °C. The remaining mass at 550 °C was 56.8%, considerably higher than for PEDOT:PSS and consistent with the high carbonaceous solid content of the formulation. The manufacturer reports a non-volatile content of 48–50% for Bectron GP 9553, determined after drying at 120 °C, which is in reasonable agreement with the large residual fraction observed by TGA, although the values should not be directly equated because the measurement conditions are different.

The stronger mass loss of the PEDOT:PSS formulation is also consistent with its nature as a low-solids content, water-based inkjet ink. According to the manufacturer, ORGACON IJ-1005 contains approximately 0.8 wt.% PEDOT:PSS in the as-supplied ink, whereas the carbon formulation is a highly concentrated screen-printing paste with 48–50% non-volatile content. Overall, the results, numerically displayed in Table 4, confirm the substantially higher inorganic/carbonaceous residue and higher-temperature stability of the screen-printable carbon formulation compared with the PEDOT:PSS ink.

**Figure 6:**
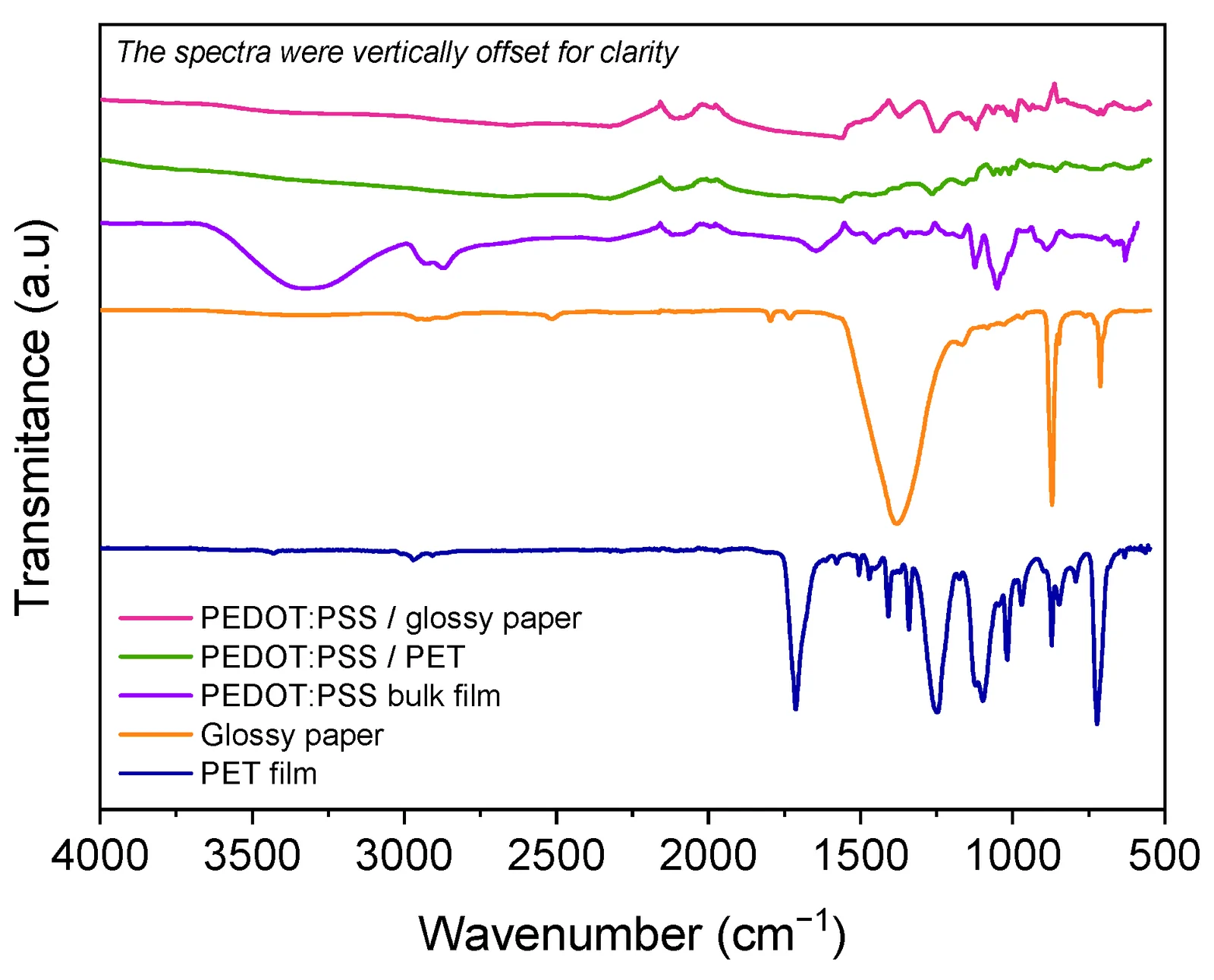
FTIR spectra of PEDOT:PSS-based films and pristine substrates.

FTIR spectroscopy was used to compare the chemical signatures of the PEDOT:PSS bulk film, PEDOT:PSS printed on PET and glossy paper, and the corresponding bare substrates (Figure 6). The PET spectrum shows the characteristic strong absorption bands of polyethylene terephthalate. The spectrum of the glossy paper is dominated by cellulose-related vibrations. The most evident difference between the PEDOT:PSS printed spectra occurs in the 3000–3600 cm^−1^ region. The PEDOT:PSS bulk film exhibits a broad absorption band centred around the O–H stretching region, which is consistent with the hydrogen-bonded water and hydroxyl-containing species associated with the hygroscopic PEDOT:PSS formulation. In contrast, this broad feature is strongly reduced or absent in the spectra of PEDOT:PSS printed on PET and glossy paper. Remarkably, the bare glossy paper also does not show a pronounced broad O–H absorption in this region. This suggests that the glossy surface coating limits the contribution of the underlying cellulose-rich paper structure to the FTIR response, which is consistent with the fact that the measurement probes primarily the surface region. The spectra of PEDOT:PSS printed on PET and glossy paper are, in fact, similar over most of the investigated spectral range. Both retain the main features associated with the PEDOT:PSS layer, particularly in the fingerprint region, while showing substantially weaker O–H-related absorption than that of the bulk PEDOT:PSS film. This similarity suggests that, despite the pronounced differences in roughness and morphology observed between PET and glossy paper by profilometry and SEM, the chemical environment detected by FTIR at the surface of the printed PEDOT:PSS layers remains broadly comparable.

The strong O–H contribution observed only in the bulk PEDOT:PSS film may indicate a higher amount of retained or associated water within the thicker PEDOT:PSS material, whereas the much thinner printed layers contain a considerably smaller detectable water contribution after drying. However, FTIR alone cannot determine whether this water is located internally within the PEDOT:PSS structure or specifically at the surface. Likewise, the spectra do not provide sufficient evidence to establish preferential surface enrichment of PEDOT or PSS. Overall, the FTIR results indicate that printing and drying substantially reduce the water-associated spectral contribution observed in the bulk PEDOT:PSS film, while the PEDOT:PSS layers printed on PET and glossy paper exhibit very similar chemical signatures. This is noteworthy, given the substantial morphological differences between the two substrates, and suggests that the substrate-dependent electrical behaviour observed in the sensors is more likely related to differences in PEDOT:PSS structure and morphology, ink penetration, interfacial interactions and environmental moisture exchange than to major changes in the chemical structure of PEDOT:PSS.

### 3.3. Electrical and Thermoresistive Properties

#### 3.3.1. Sheet Resistance and Room-Temperature Sensor Resistance

**Figure 7:**
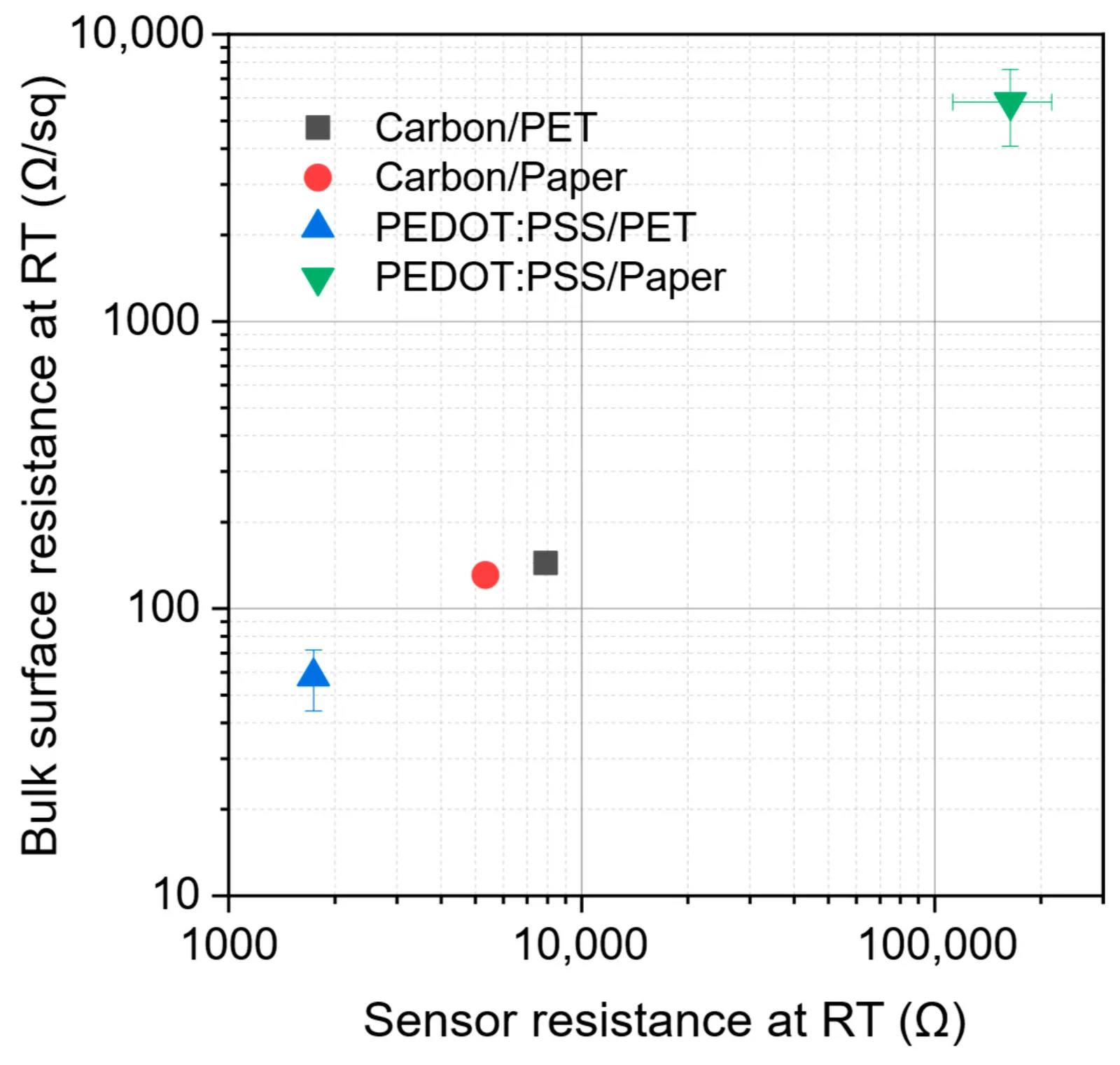
Relationship between bulk surface resistance of ink films and sensor device resistance at RT.

Four-point probe measurements revealed a strong substrate effect for PEDOT:PSS, whereas carbon showed only minor differences. Carbon sheet resistance was comparable on both PET and glossy paper (144.09 ± 1.84 and 130.65 ± 3.34 Ω/sq, respectively), while PEDOT:PSS increased from 57.93 ± 13.88 Ω/sq on PET to 5804.75 ± 1732.37 Ω/sq on glossy paper. This near two-orders-of-magnitude increase in sheet resistance is consistent with the greater sensitivity of the thin, low-viscosity inkjet-printed PEDOT:PSS layer to substrate morphology, compared with that of the thicker screen-printed carbon layer. The same trend was observed in the complete sensors, as depicted in Figure 7. PEDOT:PSS resistance increased from 1.739 ± 0.008 kΩ on PET to 163.7 ± 51.2 kΩ on glossy paper, while carbon remained within the same order of magnitude (7.90 ± 0.19 kΩ on PET and 5.34 ± 0.10 kΩ on paper). The close agreement between the sheet-resistance and device-resistance trends further supports the pronounced substrate dependence of inkjet-printed PEDOT:PSS.

#### 3.3.2. Electrical Impedance Spectroscopy

**Figure 8:**
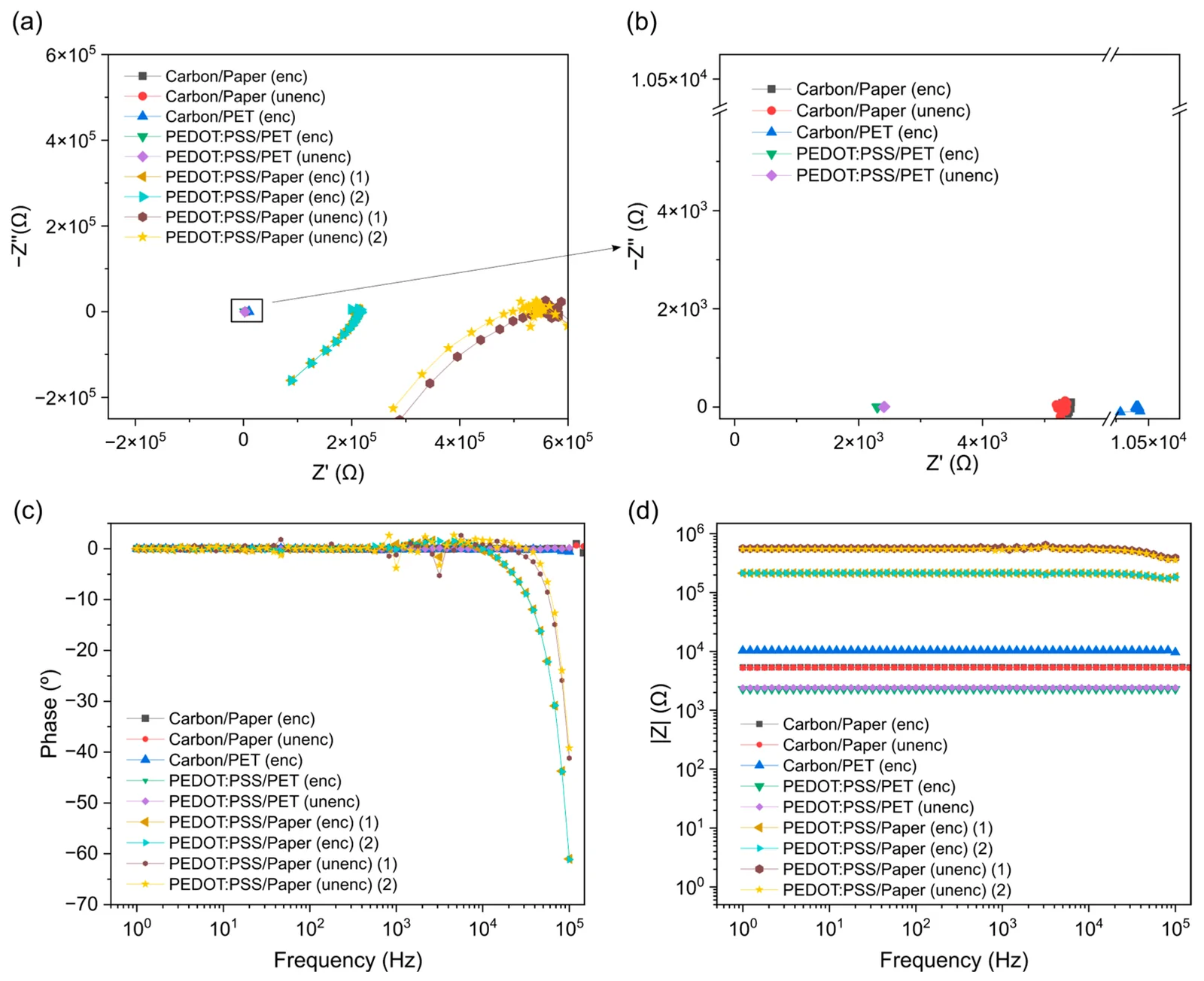
Electrical impedance spectroscopy of the printed temperature sensors. (**a**) Nyquist representation, including a magnified view of the low-impedance region (**b**), (**c**) Phase angle and (**d**) impedance magnitude as a function of frequency.

Electrical impedance spectroscopy confirmed that the printed temperature sensors were predominantly resistive over most of the investigated frequency range, as depicted in Figure 8. The Nyquist plots in (Figure 8a,b) show that the carbon-based and PEDOT:PSS/PET devices are dominated by the real component of impedance, confirming predominantly resistive behaviour. PEDOT:PSS/glossy-paper devices, however, exhibited much higher impedance and greater dispersion, consistent with the higher sheet and device resistances observed for this ink–substrate combination. Because the response was largely resistive, and the high-frequency reactive contribution could not be uniquely assigned, no equivalent-circuit fitting was performed.

The Bode phase plots (Figure 8c) further support this interpretation. Carbon and PEDOT:PSS/PET maintained phase angles close to 0°, whereas PEDOT:PSS/glossy-paper devices showed an increasingly negative phase at high frequency, indicating a growing reactive contribution. This behaviour was reproducible but was not assigned to a specific capacitive mechanism because the high impedance of these samples makes them more susceptible to parasitic and instrumental effects.

The impedance-magnitude spectra (Figure 8d) showed a nearly frequency-independent ∣Z∣ for carbon and PEDOT:PSS/PET, while PEDOT:PSS/glossy-paper displayed substantially higher impedance and a gradual decrease at high frequency. The persistent separation between PEDOT:PSS on PET and paper agrees with the four-point-probe and room-temperature resistance results, further supporting a strong substrate effect. Hence, the greater scatter observed for PEDOT:PSS on paper is consistent with a more heterogeneous conductive network resulting from the interaction of the thin inkjet-printed layer with the rougher paper substrate.

#### 3.3.3. Thermoresistive Response and TCR

**Figure 9:**
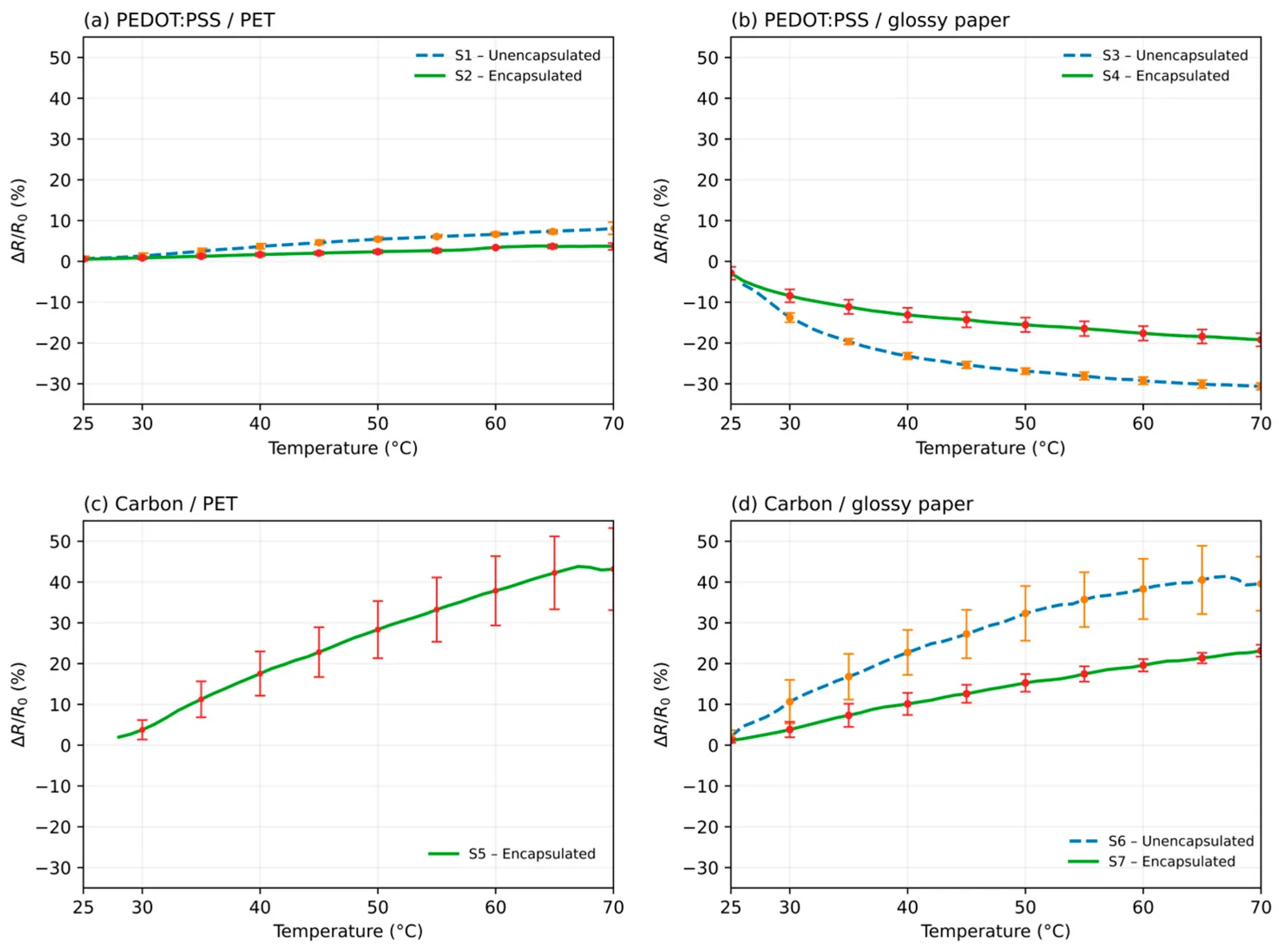
Normalised thermoresistive response of the printed temperature sensors during repeated heating cycles. Curves represent the mean ΔR/R_0_ (%) response, and error bars indicate the standard deviation across the selected heating cycles at 5 °C intervals.

The normalised heating responses and corresponding TCR values are summarised in Table 5 and Figure 9. For Figure 9, the normalised thermoresistive curves were obtained by pooling the selected heating cycles from the three independently fabricated sensors of each sensor type, and the error bars represent the standard deviation of the normalised response across those selected cycles, displayed at 5 °C intervals for clarity. In contrast, the values reported in Table 5 were calculated by first averaging the cycle-level TCR and R^2^ values for each sensor and then calculating the final mean ± standard deviation across the three independent sensors. The thermoresistive measurements were designed primarily for comparative evaluation of the different sensor configurations under standardised experimental conditions rather than for high-accuracy absolute temperature metrology. Although the use of a packaged LM35 reference sensor and hot-air heating may introduce uncertainty associated with transient thermal gradients and differences in thermal response time, the same experimental setup and procedure were maintained for all samples. The repeated heating cycles showed good reproducibility and, for most sensor configurations, high linearity, supporting the comparative interpretation of the measured TCR trends.

PEDOT:PSS sensors, in particular, exhibited opposite temperature coefficients, depending on the substrate. On PET, both unencapsulated and encapsulated devices showed PTC behaviour, with TCR values of 0.176 ± 0.012% °C^−1^ and 0.072 ± 0.006% °C^−1^, respectively. In contrast, PEDOT:PSS printed on glossy paper showed NTC behaviour, with significantly larger absolute sensitivities of −0.594 ± 0.049% °C^−1^ without encapsulation and −0.364 ± 0.017% °C^−1^ after encapsulation. This inversion in response sign further supports the strong influence of the substrate on the electrical transport of the thin inkjet-printed PEDOT:PSS layer. Moreover, the comparatively high magnitude of the NTC response observed for PEDOT:PSS on glossy paper is nevertheless consistent with previous reports showing that paper type strongly influences the thermoresistive behaviour of inkjet-printed PEDOT:PSS. For example, Barmpakos et al. reported a TCR of approximately −1.39% °C^−1^ for PEDOT:PSS printed on glossy photo paper, while Maslik et al. reported an even larger magnitude of approximately −3.0% °C^−1^ over −10 to 22 °C on a glossy photo-paper substrate [20,33]. These differences may arise from variations in surface coating, roughness, porosity, and ink penetration, which affect the morphology and connectivity of the thin PEDOT:PSS conductive network.

The carbon sensors showed PTC behaviour for all tested configurations. Carbon/PET showed a TCR of 1.168 ± 0.093% °C^−1^ in the encapsulated condition, while carbon/paper showed 1.002 ± 0.097% °C^−1^ without encapsulation and 0.510 ± 0.014% °C^−1^ after encapsulation. These results indicate a comparatively weaker substrate dependence than that observed for PEDOT:PSS within the tested configurations. However, because unencapsulated carbon/PET devices could not be reliably characterised due to mechanical failure during heating, a fully symmetric comparison of the substrate and encapsulation effects was not possible for the carbon system.

Encapsulation reduced the magnitude of the thermoresistive response for PEDOT:PSS on both substrates and for carbon on paper. For carbon/PET, only the encapsulated condition was evaluated, so the effect of encapsulation could not be independently assessed on this substrate. For PEDOT:PSS, the absolute TCR decreased by approximately 59% on PET and 39% on paper, while for carbon/paper, it decreased by about 49%. This reduction in thermoresistive response after encapsulation may result from a combination of mechanical constraint, reduced interaction with the surrounding environment, and the additional thermal resistance introduced by the polyimide encapsulation layer and its interfaces. Under transient hot-air heating, this added thermal resistance may also increase the thermal lag between the printed sensor and the adjacent LM35 reference, hence reducing the apparent TCR without necessarily indicating an equivalent intrinsic loss of sensor sensitivity. Moreover, although moisture-related effects may contribute to the observed PEDOT:PSS response, their contribution cannot be isolated in the present study because humidity was not monitored during thermal cycling. The R^2^ values further highlight differences in response quality. PEDOT:PSS/PET and carbon sensors showed high linearity, with mean R^2^ values generally above 0.97, whereas PEDOT:PSS/paper showed lower linearity, particularly in the unencapsulated condition (R^2^ = 0.841 ± 0.034). This lower linearity is consistent with the more heterogeneous morphology and electrical response observed for PEDOT:PSS on paper. The relatively small standard deviation in TCR for most conditions also indicates good cycle-to-cycle reproducibility.

## 4. Conclusions

This work demonstrated that the thermoresistive behaviour of printed temperature sensors cannot be considered independently of the substrate and fabrication process. The strongest substrate effect was observed for inkjet-printed PEDOT:PSS. The considerably rougher and more heterogeneous glossy-paper surface produced a less uniform conductive layer than did PET, as supported by profilometry, SEM, EDS and electrical characterisation. Accordingly, PEDOT:PSS sheet resistance increased by approximately two orders of magnitude from PET to paper, in agreement with the corresponding increase in complete-device resistance.

Moreover, the substrate also altered the sign of the PEDOT:PSS temperature response. PEDOT:PSS/PET sensors exhibited PTC behaviour, with a TCR of +0.176 ± 0.012% °C^−1^, whereas PEDOT:PSS/paper showed NTC behaviour, with a substantially larger absolute TCR of −0.594 ± 0.049% °C^−1^. Electrical impedance measurements confirmed predominantly resistive behaviour for all devices over most of the investigated frequency range, while the paper-supported PEDOT:PSS sensors exhibited greater impedance dispersion and variability, consistent with their heterogeneous electrical response.

In comparison, the thicker screen-printed carbon layers were considerably less sensitive to substrate morphology. Carbon sensors maintained PTC behaviour on both PET and glossy paper, with TCR values of +1.168 ± 0.093% °C^−1^ and +1.002 ± 0.097% °C^−1^, respectively. Encapsulation reduced the magnitude of the temperature response for PEDOT:PSS on both substrates and for carbon on glossy paper, indicating that mechanical constraint and reduced environmental interaction can influence thermoresistive sensitivity.

The present study was restricted to the 25–70 °C range to enable comparison of the different sensor configurations while minimizing thermomechanical effects associated with the PET substrate. Low-temperature operation was not investigated and would require controlled temperature and humidity conditions, particularly for PEDOT:PSS- and paper-based devices, where moisture uptake and condensation may influence the electrical response. Since humidity and water content were not directly monitored during thermal cycling, moisture-related effects are considered a plausible contributing factor rather than a confirmed mechanism. Future work should therefore extend the characterisation to broader temperature ranges using suitable substrates and controlled environmental conditions. In addition, thinner encapsulation strategies, such as polymer membranes, screen-printed dielectric coatings with improved thermal conductivity, or very thin spray- or spin-coated protective layers, should be explored to reduce thermal lag while maintaining mechanical and environmental protection.

Overall, the results show that substrate selection can substantially modify both the electrical properties and even the sign of the thermoresistive response of thin printed conductive layers, particularly for inkjet-printed PEDOT:PSS. In contrast, thicker screen-printed carbon layers showed a more consistent PTC response across the tested substrates, although the absence of an unencapsulated carbon/PET condition limits a fully symmetric assessment of substrate and encapsulation effects. These findings highlight the importance of considering the complete ink–substrate–printing–encapsulation system when designing flexible printed temperature sensors.

## Figures and Tables

**Table 2:** Characteristics of the inks used in this work, obtained from their respective technical datasheets.

Test	Properties	
	Agfa Orgacon^TM^ IJ-1005	Elantas Bectron^®^ GP 9553
Type	Water-based PEDOT:PSS	Carbon Nanoparticles
Solids (%)	0.8	48–50
Viscosity (mPa.s)	11–14	10,000–20,000
Density (g/cc)	0.98–1.00	1.1–1.3
Coverage (cm^2^/g)	200–500	1400–1600
Dried Print Thickness (µm)	0.05–0.3	5–10
Resistivity (Ω/sq)	75–800	50
Shelf life (months)	6	6
Surface tension (mN/m)	31–34	Not Available

**Table 3:** Printed sensors produced and evaluated in this work.

Sensor Type	Ink	Substrate	Encapsulation	Technique	Sensor Design
S1	PEDOT:PSS	PET	No	Inkjet printing	Meander 1
S2	PEDOT:PSS	PET	Yes (PI tape)	Inkjet printing	Meander 1
S3	PEDOT:PSS	Paper	No	Inkjet printing	Meander 1
S4	PEDOT:PSS	Paper	Yes (PI tape)	Inkjet printing	Meander 1
S5	Carbon	PET	Yes (PI tape)	Screen printing	Meander 2
S6	Carbon	Paper	No	Screen printing	Meander 2
S7	Carbon	Paper	Yes (PI tape)	Screen printing	Meander 2

**Table 4:** Thermal properties of inks used in the printed sensors.

Ink	T1 (°C)	T2 (°C)	T3 (°C)	Residuals (%)
PEDOT:PSS (Agfa)	174.8	320.3	382.4	14.95
Carbon (Elantas)	141.8	436.2	-	56.8

**Table 5:** Thermoresistive performance of the printed temperature sensors, including TCR, linearity, and response type.

Sensor Type	Ink	Substrate	Encap.	TCR (% °C^−1^)	SD	R^2^	SD	Response Type
S1	PEDOT:PSS	PET	No	0.176	0.012	0.985	0.010	PTC
S2	PEDOT:PSS	PET	Yes	0.072	0.006	0.993	0.004	PTC
S3	PEDOT:PSS	Paper	No	−0.594	0.049	0.841	0.034	NTC
S4	PEDOT:PSS	Paper	Yes	−0.364	0.017	0.917	0.009	NTC
S5	Carbon	PET	Yes	1.168	0.093	0.995	0.008	PTC
S6	Carbon	Paper	No	1.002	0.097	0.974	0.010	PTC
S7	Carbon	Paper	Yes	0.510	0.014	0.994	0.002	PTC

## Data Availability

The raw data supporting the conclusions of this article will be made available by the authors on request.
